# Long-Term Impact of Diagnosed Fetal Anomaly on Parental Traumatic Stress, Resilience, and Relationship Satisfaction

**DOI:** 10.1093/jpepsy/jsac085

**Published:** 2022-11-18

**Authors:** Aurora Oftedal, Mona Bekkhus, Guttorm Haugen, Odin Hjemdal, Nikolai Olavi Czajkowski, Anne Kaasen

**Affiliations:** Faculty of Health Sciences, Oslo Metropolitan University, Oslo, Norway; Promenta Research Center, Department of Psychology, University of Oslo, Oslo, Norway; Division of Obstetrics and Gynaecology, Oslo University Hospital, Oslo, Norway; Faculty of Medicine, Institute of Clinical Medicine, University of Oslo, Oslo, Norway; Department of Psychology, Norwegian University of Science and Technology (NTNU), Trondheim, Norway; Promenta Research Center, Department of Psychology, University of Oslo, Oslo, Norway; Department of Mental Disorders, Norwegian Institute of Public Health, Norway; Faculty of Health Sciences, Oslo Metropolitan University, Oslo, Norway

**Keywords:** congenital malformation, long-term, prenatal diagnosis, psychological adjustment, relationship satisfaction, resilience, traumatic stress

## Abstract

**Objective:**

Knowledge regarding the long-term psychological adjustment of parents to children with prenatal diagnosis of congenital malformation is scarce. The aim of this study is to examine traumatic stress trajectories, resilience, and relationship satisfaction among parents to children with prenatal diagnosis of a congenital malformation, and to compare this to a sample of non-affected parents.

**Methods:**

A prospective longitudinal cohort study was conducted at a tertiary perinatal referral center. Ninety-three mothers and 80 fathers who received a diagnosis of fetal anomaly during obstetric ultrasound examination (study group), and 110 mothers and 98 fathers with normal ultrasound findings (comparison group), reported their traumatic stress at four timepoints during pregnancy (T1–T4), 6 weeks after birth (T5), and 10–12 years after birth (T6). Resilience and relationship satisfaction was reported at 10–12 years after birth.

**Results:**

Parents to children with a congenital malformation experienced significantly elevated traumatic stress levels over time, compared with parents of children without congenital malformation. The difference between groups was largest acutely after diagnosis and remained significant 10–12 years after the birth of the child. Resilience and relationship satisfaction levels were similar in both groups.

**Conclusions:**

Despite experiencing high levels of traumatic stress over time, parents to children with a congenital malformation reported resilience and relationship satisfaction at similar levels to non-affected parents. This suggests that despite ongoing long-term distress, parents are still able to maintain positive psychological coping resources.

## Introduction

Fetal anomaly is a genetic or physical condition that affects the embryo or fetus and can vary from minor malformations to severe conditions than may lead to death or stillbirth (e.g., heart defects, cleft lip, neural tube defects, and Down’s syndrome). It is diagnosed in 2–5% of all pregnancies (Dolk et al., 2010). Approximately half of all anomalies are detectable during weeks 18–22 of pregnancy (Syngelaki et al., 2019). Although some women are at high risk, the majority occur in low-risk groups, thus the diagnosis is most often unexpected (Asplin et al., 2015; Denney-Koelsch et al., 2015; Kowalcek, 2007). For expectant parents, receiving a diagnosis of fetal anomaly is a highly stressful and potentially traumatic event that can elicit immediate feelings of grief, loss, anger, depression, and anxiety (Aite et al., 2011). Several studies have documented elevated levels of traumatic stress symptoms among expectant parents to children with congenital malformations in the weeks following diagnosis (Cole et al., 2016; Kaasen et al., 2013, 2017). For many parents, distress remains elevated and stable for several months after the birth of the child (Bevilacqua et al., 2018; Brosig et al., 2007), although one study found a reduction in traumatic stress over the first 12 months (Ellens et al., 2017). Beyond the first year, however, little is known about the long-term consequences of diagnosis of fetal anomaly on parental traumatic stress levels.

According to the Model of Pediatric Medical Traumatic Stress (Kazak et al., 2006), traumatic stress symptoms (e.g., arousal, intrusion, and avoidance) are common responses to a child’s medical event and can be divided into three phases. Phase I includes the diagnosis and acute aftermath, phase II includes the early and evolving reaction to the event, and phase III includes long-term traumatic stress responses. The timing and duration of each phase will vary, but during phase I, nearly all parents will experience acute traumatic stress responses (Alderfer & Kazak, 2006). As the immediate reaction to the diagnosis begins to resolve, parents may begin to return to normal functioning, but the demands and ramifications of the diagnosis can give rise to ongoing distress.

In addition to diagnosis being a single traumatic event, having a child with a congenital malformation can be a complex and prolonged stressful situation that continue to affect the parents’ emotional and social wellbeing (Cole et al., 2016; Skreden et al., 2010). Previous research has found that parents of children with chronic diseases or disabilities experience increased psychological distress and anxiety compared with parents of healthy children (Brehaut et al., 2004; Rozensztrauch et al., 2020; Theule et al., 2013; Werner et al., 2019). Caring for a child with a chronic health condition can include both daily and long-term stressors such as management of the child’s medical regiment; worry regarding the child’s health condition and quality of life; uncertainty about the child’s current and future independence; and increased organizational, financial, and emotional burdens compared with typical parenting responsibilities (Brehaut et al., 2004; Goldbeck & Melches, 2006).

The ability to maintain or regain emotional health in the face of adversity may be understood through the construct of resilience. Resilience is a complex construct describing collections of protective factors within the individual and their environment, such as positive psychological dispositional attributes, family support and cohesion, and external support systems (Bonanno, 2004; Friborg et al., 2003). In the current study, resilience pertains to the protective personal and psychosocial resources of adults in otherwise normal circumstances who are exposed to a highly disruptive event (Bonanno, 2004).

While resilience may be considered a buffer of psychopathology in response to crisis (Friborg et al., 2006; van der Walt et al., 2014), resilience can also be thought of as an outcome that in itself is affected by adversity (Gavidia‐Payne et al., 2015; Heiman, 2002). Exposure to crisis has in some instances been associated with increased resilience (Scali et al., 2012), a form of post-traumatic growth (Dickinson, 2021). However, exposure to trauma may also diminish resilience when it leads to a sense of isolation, hopelessness, or perceived inability to cope (Rosenberg et al., 2014). Understanding how different factors drive variations in resilience and growth from adversity remains an area of ongoing investigation. To our knowledge, no previous research has examined the impact of diagnosis of fetal anomaly on parental resilience.

It has been argued that parental traumatic stress can interfere with parenting practices and bear adverse family outcomes such as increased conflict and reduced relationship satisfaction (Bryant et al., 2018; McDonald et al., 2011; Suttora et al., 2014; Wamser-Nanney & Sager, 2020). Family resilience theory (Van Schoors et al., 2015) makes the assumption that a family is more than the sum of its parts (Bertalanffy, 1968), and that a serious diagnosis not only affects the individuals within the family, but also their relationships with one another (Alderfer & Kazak, 2006). It is therefore important to understand the impact of diagnosis of fetal anomaly on parents’ relationships with one another. Within the framework of family resilience theory, relationship satisfaction and resilience are interconnected, as cohesion and social support are important aspects of family resilience (Bradley & Hojjat, 2017; Hou & Ng, 2014). Consistent with this, high levels of parental conflict may limit the parents’ ability to meet the potentially substantial demands of caring for a child with a congenital disorder (Ludman, 2003). Importantly, children with malformations might already have compromised health and development. The risk of exposure to negative family dynamics may further threaten the health outcome for these children (Hastings, 2002). Understanding more about the impact of congenital malformations on family harmony and parents’ psychosocial health may help us improve support to families that could be vulnerable to elevated parental distress and conflict.

Most previous studies on parental wellbeing in the context of children’s illness or disability focus on maternal distress, although fathers are increasingly involved in childrearing (Redshaw & Henderson, 2013). Consequently, there is little knowledge regarding paternal distress following diagnosis of a congenital malformation. To our knowledge, only two studies have examined the long-term impact of the birth of a child with prenatal diagnosis of fetal anomaly on maternal and paternal traumatic stress (Cole et al., 2016; Skreden et al., 2010). Cole et al. (2016) reported that parents experienced elevated levels of distress with men reporting lower symptoms of posttraumatic stress, anxiety, and depression than women. However, this study retrospectively examined symptoms 2 years after the diagnosis, thus findings are subject to recall bias. Skreden et al. (2010) found that mothers and fathers showed similar trajectories over time, but with mothers reporting significantly higher distress levels. However, neither of these previous studies included a comparison group of non-affected parents; thus, it is difficult to assess now the challenges of parenting a child with congenital malformation compared with that of parenting in general. Furthermore, none of these studies included other aspects of adjustment, such as resilience and relationship satisfaction.

With the current study, there were three main aims. The first aim was to describe the development of traumatic stress in mothers and fathers of children with congenital malformations over time, from pregnancy until 10–12 years after birth. We compared this with traumatic stress trajectories in non-affected parents. Secondly, we aimed to explore resilience among parents with and without children with congenital malformations at 10–12 years after birth. We did this by comparing resilience among mothers and fathers in the study and comparison group and examine the association between resilience and traumatic stress at 10–12 years after birth. Lastly, the third aim was to compare relationship satisfaction among men and women in the study and comparison group at 10–12-year follow-up, and to examine the correlations between relationship satisfaction, resilience, and traumatic stress.

## Methods

### Study Design and Participants

The present study is part of a larger, ongoing longitudinal study examining parental stress reactions following the detection of fetal anomalies (the SOFUS study). Participant recruitment occurred among pregnant women and their partners receiving obstetric care at Oslo University Hospital—Rikshospitalet. Participants in the study group were recruited following the identification of a suspected structural fetal anomaly during obstetric ultrasound examination. In the comparison group, participants were recruited following normal findings on routine ultrasound scan. The initial sample consisted of 330 expectant parents of 180 fetuses with a detected malformation (*n *=* *180 pregnant women, *n *=* *150 partners), and 211 expectant parents of 111 fetuses with normal ultrasound findings and an uncomplicated pregnancy history (*n *=* *111 pregnant women, *n *=* *100 partners). All the partners were male. Within the study group, 87 of 180 women terminated the pregnancy following diagnosis, and these participants are not included in the current study. Women who were under the age of 18 years, who could not read or write in Norwegian, or who were not legally competent to provide consent were not included in the study. Partners were considered eligible if the pregnant woman met inclusion criteria and they were legally competent to provide consent. Further details regarding enrollment have been published elsewhere (Bekkhus et al., 2020; Kaasen et al., 2017).

### Procedure

Recruitment and reporting of traumatic stress took place within 72 hr of either a diagnosis of fetal anomaly or normal ultrasound findings (T1). Traumatic stress was collected 3 more times during pregnancy: 2–3 weeks after inclusion (T2), at 30 weeks gestation (T3) and at 36 weeks gestation (T4). Postnatal traumatic stress was collected 6 weeks after birth (T5). Data collection occurred between May 2006 and February 2009 for T1–T5.

For the 10–12-year follow-up, participants were contacted by letter during October 2019 and asked to complete an online questionnaire. If they did not respond within 4–6 weeks, they were contacted one more time by phone and/or text message and reminded of the invitation to participate. Effort to locate families that had been lost to follow-up was made using the National Population Register (The Norwegian Tax Administration, 2019) and by searching the Internet. Parents to children that had died (*n* = 6) were not invited to participate due to ethical concerns. The follow-up questionnaire included questions about traumatic stress, as well as resilience and relationship satisfaction. Participants provided their responses without remuneration or compensation.

### Measures

Sociodemographic information, including age, education, number of children, and marital status, as well as severity of fetal diagnosis, was collected at inclusion (T1) and T6. Clinical study variables such as prenatal diagnosis and gestational age were assessed at time of inclusion using electronic medical charts. During pregnancy the severity of diagnoses was categorized as lethal or serious (e.g., ventriculomegaly), or mild to moderate (e.g., cleft lip/palate). The process for categorization of severity has been described in Kaasen et al. (2010). Data on race and ethnicity were not collected due to the ethnic homogeneity of the sample.

#### Impact of Event Scale

Traumatic stress was measured using the Impact of Event Scale (IES) (Horowitz et al., 1979; Weiss, 2007). The IES is a 22-item questionnaire measuring emotional and behavioral symptoms over the past week in response to a defined stressful or traumatic event. In this case, the questions were asked with reference to “your child’s condition.” The original IES contains two subscales measuring intrusion (disturbing affects and thoughts about the traumatic event) and avoidance (effortful attempts at avoiding thoughts and images related to the event). The IES version used in this study includes six additional items measuring arousal (i.e., irritability, difficulty concentrating, and hypervigilance) and one additional item measuring intrusion, as published by Weiss (2007). Items were scored from 0 to 5, with 0 meaning “not at all” and 5 meaning “often.” The scale has been validated for use with Norwegian populations with a Cronbach’s alpha of 0.85, 0.84, and 0.62 for the intrusion, avoidance, and arousal subscales, respectively (Eid et al., 2009). At T1, Cronbach’s alpha for the measure was 0.88–0.92 for the various subscales among women, and 0.85–0.91 for the various subscales among men.

#### Resilience Scale for Adults

Resilience was measured only at T6, using the Resilience Scale for Adults (RSA) (Friborg et al., 2003). The RSA consists of 33 items and was developed to measure inter- and intrapersonal resources that may facilitate adaptation to adverse life events. It is comprised of six factors: Positive perception of self (six items), positive perception of the future (four items), social competence (six items), structured style (four items), family cohesion (six items), and social resources (seven items). The first four factors make up the intrapersonal resources, while the last two make up the interpersonal resources. Items are scored on a 7-point scale with lower numbers indicating less resilience. Total score ranges from 33 to 231. The measure has been validated for use in Norwegian with a Cronbach’s alpha of 0.93 for the total score (Friborg et al., 2003). In the current sample, Cronbach’s alpha for the total RSA was 0.95 among women and 0.93 among men.

#### Relationship Satisfaction Scale

Relationship satisfaction was measured at T6 using the Relationship Satisfaction scale (RS) (Røysamb et al., 2014). The RS consists of 10 statements that are answered on a 6-point scale ranging from 1—completely disagree to 6—completely agree. Total scores range from 10 to 60, where lower scores indicate less relationship satisfaction. The scale has been validated in Norwegian with a Cronbach’s alpha of 0.92 (Røysamb et al., 2014). Cronbach’s alpha for the measure in our study was 0.93 among women and 0.92 among men.

### Statistical Analysis

The power to detect a difference in traumatic stress among mothers and fathers with and without diagnosis of fetal anomaly was computed using R version 4.0.4 (R foundation for Statistical Computing, Vienna, Austria) with the package “pwr.” We estimated the expected effect size using data from Skreden et al. (2010) in comparison to population norms for the IES in a Norwegian adult population (Heir et al., 2010). It was found that a total sample size of *n* = 24 mothers and *n* = 30 fathers would give *β *= 0.90 to detect a significant effect with *α *= 0.05.

Linear mixed models were fitted to test whether diagnosis of fetal anomaly was significantly related to change in traumatic stress scores over time. The models were calculated using R version 4.0.4 (R foundation for Statistical Computing, Vienna, Austria) with the package “lme4” (Bates, 2010) and *α* = 0.05. Missing data were handled using listwise deletion. Model parameters were then estimated by means of maximum likelihood, an approach that makes use of all observed data. Competing models were compared using the likelihood ratio test (Crainiceanu & Ruppert, 2004). Since a group mean can conceal changes on an individual level, individual trajectories, as well as means and 95% confidence intervals (CIs) were included in the graphs.

Independent sample *t*-tests were used to examine the difference in resilience and relationship satisfaction between the study and comparison group at T6. Paired sample *t*-tests were used to examine differences between men and women in resilience and relationship satisfaction. The relationship between traumatic stress and resilience among men and women in the study group and the comparison group was examined using simple linear regression equations, with resilience at T6 regressed onto intrusion, avoidance, and arousal at T6. Linear regression was also used to examine the potential influence of demographic factors such as age and education on resilience. The relationship between relationship satisfaction, resilience, and traumatic stress was examined using Pearson’s correlation. The *t*-tests, regression equations, and correlations were calculated using IBM SPSS version 27 (Statistical Package for the Social Sciences, IBM, Armonk, NY, USA).

### Ethics

The study was approved by the Regional Committee for Medical Research Ethics, Southern Norway, Oslo, Norway, on December 21, 2005 (reference number S-05281) and the 10–12-year follow-up was approved on May 10, 2016 (reference number: 2016/776/REK). All participants gave their written informed consent prior to participation.

## Results

### Descriptive Data

The sociodemographic characteristics of the participants and severity of fetal diagnosis at inclusion (T1) and 10–12-year follow-up (T6) are shown in Table 1. At T1, mothers in the study group were significantly younger, had fewer years of education, and at T6, they were more likely to have only one than mothers in the comparison group. Among fathers there was a significant difference in education at T1. Within the study group, fetal anomalies included all types of diagnoses, but the most common were cleft lip/palate (9%), gastroschisis (9%), unilateral or bilateral hydronephrosis (6%), intraabdominal cyst (6%), heart defect (5%), ventriculomegaly (5%), and talipes equinovarus (clubfoot; 4%).

**Table I.** Characteristics of mothers and fathers in the study group (diagnosed fetal anomaly) and comparison group (no diagnosis of fetal anomaly) at inclusion (T1) and follow-up (T6; 10–12 years after birth)

	Mothers			Fathers		
	Study group *N* (%)	Comparison group *N* (%)	*p*-value	Study group *N* (%)	Comparison group *N* (%)	*p*-value
Age at T1			**.015**			.058
Mean (SD)	29.59 (4.72)	31.64 (4.16)		32.52 (5.58)	33.91 (4.67)	
Married or cohabiting at T1			.401			.999
Yes	90 (99%)	111 (100%)		80 (100%)	98 (100%)	
No	1 (1%)	0 (0%)		0 (0%)	0 (0%)	
Married or cohabiting at T6			.150			.711
Yes	26 (92%)	38 (88%)		15 (94%)	36 (97%)	
No	2 (8%)	5 (12%)		1 (6%)	1 (3%)	
Education (T1)			**<.001**			**<.001**
High school or less	39 (42%)	18 (16%)		39 (48%)	15 (15%)	
Some college or more	52 (57%)	93 (84%)		41 (51%)	83 (85%)	
Education (T6)			**.014**			.217
High school or less	8 (29%)	4 (9%)		5 (31%)	3 (8%)	
Some college or more	20 (71%)	39 (91%)		11 (69%)	34 (92%)	
Number of children prior to index child (T1)			.362			
0	13 (46%)	24 (56%)		N/A	N/A	
1 or more	15 (54%)	19 (44%)		N/A	N/A	
Number of children at follow-up (T6)			**.005**			.948
1	13 (46%)	5 (11%)		4 (25%)	8 (22%)	
2	10 (35%)	26 (60%)		8 (50%)	19 (51%)	
3 or more	5 (17%)	10 (23%)		4 (25%)	9 (24%)	
Severity of fetal anomaly (T1)						
Lethal or serious	47 (51%)			36 (45%)		
Mild to moderate	46 (49%)			44 (55%)		
Severity of fetal anomaly (T6)						
Lethal^a^ or serious	16 (57%)			10 (62%)		
Mild to moderate	12 (43%)			6 (38%)		

*Notes*. Groups were compared using chi-square tests and independent samples *t*-tests as appropriate. Significant differences are highlighted in bold.

a None of the parents with a lethal diagnosis participated at T6.

### Attrition

Attrition through pregnancy has been reported previously (Bekkhus et al., 2020; Kaasen et al., 2017). At 6 weeks postpartum (T5), traumatic stress scores were missing for 11% of the sample (15% in the study group and 9% in the comparison group). At 10–12-year follow-up (T6), traumatic stress scores were missing for 60% of the sample (63% in the study group and 58% in the comparison group). We found no significant differences among those with missing scores at T6 and those without.

### Traumatic Stress Trajectories Among Mothers

**Figure 1.**
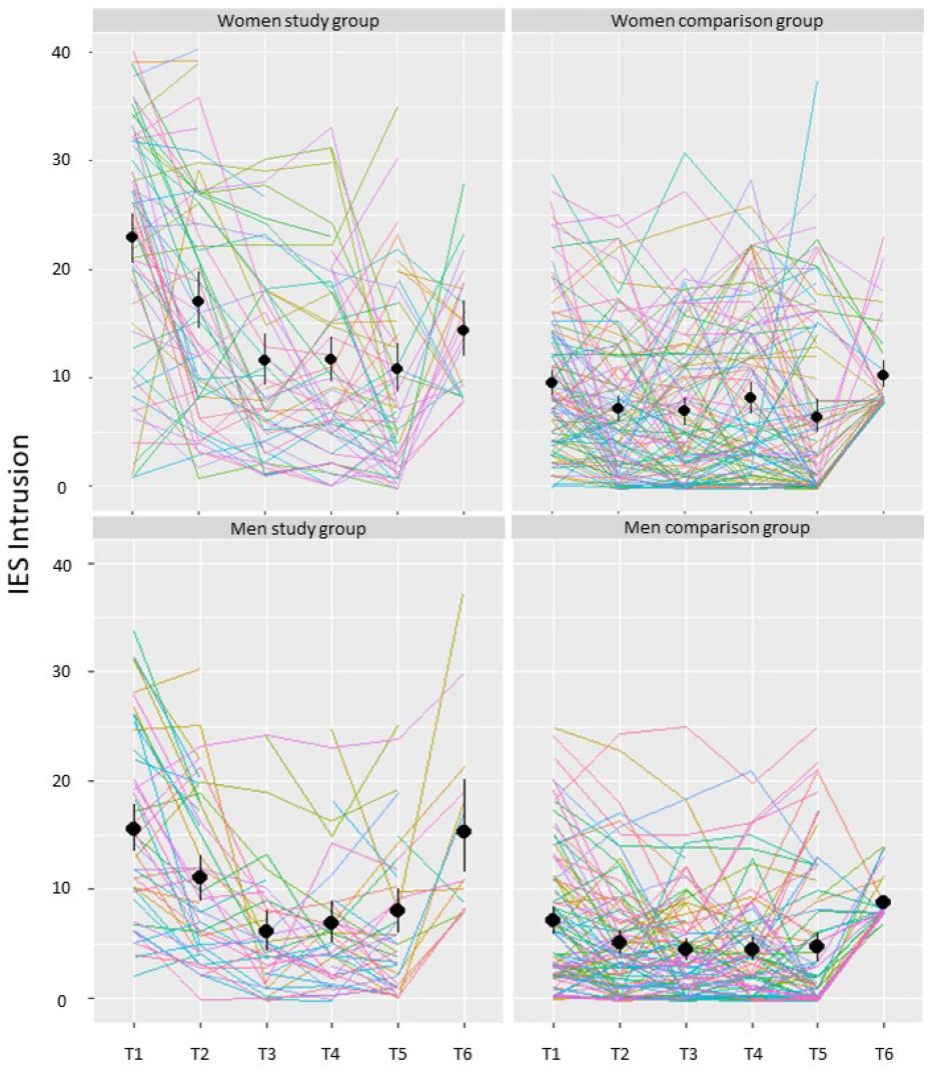
Impact of Events Scale (IES) subscale intrusion among parents in the study and comparison group from time during pregnancy (T1–T4), 6 weeks after birth (T5), until 10–12 years birth (T6). The black circles with a line indicate the group mean and 95% CI at each timepoint. The colored lines indicate individual trajectories.

A series of linear mixed model analyses with maximum-likelihood tests were performed to examine intrusion, avoidance, and arousal over time among mothers in the study and comparison group. Time was entered as a fixed effect in Model 1, along with a random effect for each participant. Independent of group, time had a significant effect on all subscales of traumatic stress: intrusion, *χ*^2^(5) = 144.92, *p* < .001; avoidance, *χ*^2^(5) = 249.55, *p* < .001; and arousal, *χ*^2^(5) = 129.77, *p* < .001. In Model 2, time and group were entered as fixed effects, along with an interaction effect of time and group. Model 2 fits significantly better than Model 1 for all subscales of traumatic stress. There was a significant effect of group such that mothers in the study group experienced more traumatic stress than mothers in the comparison group: intrusion, *χ*^2^(1) = 71.43, *p* < .001; avoidance: *χ*^2^(1) = 73.21, *p* < .001; and arousal: *χ*^2^(1) = 47.88, *p* < .001. There was also a significant interaction between time and group: intrusion, *χ*^2^(5) = 85.79, *p* < .001; avoidance: *χ*^2^(5) = 39.08, *p* < .001; and arousal, *χ*^2^(5) = 104.25, *p* < .001. The mean levels of traumatic stress at each timepoint are shown in Table II and further details on the linear mixed models are shown in Supplementary Table 4 (Supplementary Materials). Figure 1 illustrates traumatic stress trajectories for intrusion.

**Table II.** Traumatic stress levels, resilience, and relationship satisfaction among mothers and fathers in the study group (diagnosed fetal anomaly) and comparison group (no diagnosis of fetal anomaly)^a^

	Mothers			Fathers		
	Study group	Comparison group	*p*-value	Study group	Comparison group	*p*-value
	*M* (SD)	*M* (SD)		*M* (SD)	*M* (SD)	
Traumatic stress (T1)	*N* = 93	*N* = 110		*N* = 80	*N* = 98	
Intrusion	22.63 (10.25)	9.49 (6.60)	**<.001**	15.06 (9.52)	7.07 (6.22)	**<.001**
Avoidance	10.36 (8.23)	2.45 (4.05)	**<.001**	7.16 (7.02)	1.68 (2.92)	**<.001**
Arousal	12.06 (8.09)	3.68 (4.25)	**<.001**	6.35 (5.83)	2.21 (2.46)	**<.001**
Traumatic stress (T2)	*N* = 69	*N* = 104		*N* = 53	*N* = 95	
Intrusion	17.22 (10.74)	7.13 (6.41)	**<.001**	11.13 (7.67)	5.09 (5.45)	**<.001**
Avoidance	7.72 (7.90)	1.32 (2.51)	**<.001**	4.98 (5.91)	1.03 (1.81)	**<.001**
Arousal	8.58 (7.85)	2.96 (3.79)	**<.001**	4.92 (5.08)	1.59 (1.89)	**<.001**
Traumatic stress (T3)	*N* = 53	*N* = 108		*N* = 40	*N* = 95	
Intrusion	11.83 (8.69)	6.91 (6.81)	**<.001**	6.28 (6.01)	4.43 (4.91)	.065
Avoidance	5.08 (6.94)	1.38 (3.28)	**<.001**	3.18 (4.85)	0.83 (2.15)	**<.001**
Arousal	6.60 (6.35)	3.53 (3.72)	**.002**	2.98 (3.62)	1.65 (1.97)	**.007**
Traumatic stress (T4)	*N* = 63	*N* = 103		*N* = 49	*N* = 83	
Intrusion	12.05 (9.23)	8.09 (7.51)	**.003**	7.31 (6.90)	4.43 (4.95)	**.006**
Avoidance	5.46 (7.54)	1.04 (2.48)	**<.001**	3.14 (4.23)	0.75 (1.90)	**<.001**
Arousal	6.57 (6.83)	4.17 (4.17)	**.007**	3.06 (3.83)	2.07 (2.87)	.094
Traumatic stress (T5)	*N* = 80	*N* = 103		*N* = 60	*N* = 88	
Intrusion	11.18 (9.40)	6.39 (7.97)	**<.001**	8.25 (7.80)	4.67 (6.31)	**.003**
Avoidance	5.74 (7.37)	0.70 (2.23)	**<.001**	3.65 (5.60)	0.83 (2.25)	**<.001**
Arousal	5.49 (6.10)	3.14 (3.68)	**.003**	4.37 (5.58)	2.19 (2.45)	**.002**
Traumatic stress (T6)	*N* = 28	*N* = 43		*N* = 16	*N* = 37	
Intrusion	14.14 (6.90)	10.23 (3.97)	**.003**	14.81 (8.61)	8.78 (1.94)	**<.001**
Avoidance	11.50 (7.78)	8.51 (1.62)	**.017**	11.06 (4.89)	8.36 (1.15)	**.003**
Arousal	9.79 (4.86)	8.51 (3.67)	.214	9.69 (5.12)	8.47 (2.93)	.283
Resilience (T6)	*N* = 28	*N* = 42		*N* = 16	*N* = 37	
Total	165.56 (28.22)	174.83 (19.01)	.113	181.13 (30.02)	183.51 (21.71)	.745
Interpersonal	71.64 (11.46)	73.79 (6.79)	.324	73.00 (14.05)	76.16 (9.01)	.330
Intrapersonal	96.48 (17.95)	100.63 (13.72)	.279	108.13 (4.48)	107.35 (14.01)	.666
Relationship satisfaction (T6)	*N* = 26	*N* = 41		*N *= 16	*N* = 36	
Total	50.08 (9.57)	52.00 (6.24)	.161	51.38 (7.27)	50.00 (6.72)	.857

*Note.* T1 = Time 1 (inclusion); T2 = Time 2 (2–3 weeks after T1); T3 = Time 3 (gestational age 30 weeks); T4 = Time 4 (gestational age 36 weeks); T5 = Time 5 (6 weeks postpartum); T6 = Time 6 (10–12 years after birth).

a Groups were compared using independent samples *t*-tests. Significant differences are highlighted in bold.

### Traumatic Stress Trajectories Among Fathers

The models were repeated using intrusion, avoidance, and arousal scores among fathers in the study and comparison group. In Model 1, time had a significant effect on all subscales of traumatic stress: intrusion, *χ*^2^(5) = 173.83, *p* < .001; avoidance, *χ*^2^(5) = 413.26, *p* < .001; and arousal, *χ*^2^(5) = 321.69, *p* < .001. Model 2 fits significantly better than model 1 for all subscales of traumatic stress. In Model 2, there was a significant effect of group on all subscales of traumatic stress: intrusion, *χ*^2^(1) = 35.85, *p* < .001; avoidance, *χ*^2^(1) = 49.02, *p* < .001; and arousal, *χ*^2^(1) = 32.95, *p* < .001. In addition, there was a significant time × group interaction: intrusion, *χ*^2^(5) = 44.74, *p* < .001; avoidance, *χ*^2^(5) = 35.31, *p* < .001; and arousal, *χ*^2^(5) = 33.89, *p* < .001.

### Resilience at 10–12-Year Follow-Up

Resilience levels were similar among mothers in the study group and the comparison group, *t*(65) = −1.61, *p* = .11, and 95% CI [−20.79, 2.24]. Similarly, resilience levels were similar among men in the study group and the comparison group, *t*(51) = −0.33, *p* = .75, and 95% CI [−17.08, 12.30]. Looking at separate factors of resilience, there were no significant group differences in any of the interpersonal or intrapersonal factors (see Table II for partial results). Age and education did not significantly predict resilience.

Men and women’s resilience correlated within couples, Pearson’s *r* = .68, *p* < .001. However, among the couples where both the man and the woman participated (*n* = 37) there was a significant difference between men and women, such that men reported more resilience (*M *=* *185.56, *SD *=* *22.76) than women (*M *=* *174.47, *SD *=* *22.76), *t*(36) = 3.27, *p* < .01, and 95% (CI) [3.90, 16.37]. More specifically, men reported higher resilience in terms of intrapersonal factors (*M *=* *110.67, *SD *=* *13.87), than women (*M *=* *101, *SD *=* *14.94), *t*(36) = 4.17, *p* < .001, and 95% CI [4.72, 13.67]. Men and women were similar in terms of interpersonal factors, *t*(36) = .99, *p* = .33, and 95% CI [−4.61, 1.59].

### The Relationship Between Traumatic Stress and Resilience at 10–12-Year Follow-Up

Among women in the study group, resilience was inversely related to traumatic stress: IES intrusion: *b* = −0.12 and 95% CI [−0.22, −0.02]; IES avoidance: *b* = −0.13 and 95% CI [−0.24, −0.02]; and IES arousal: *b* = −0.094 and 95% CI [−0.16, −0.03]. Resilience and traumatic stress were not significantly related among women in the comparison group.

Among men in the study group there was an inverse relationship between arousal and resilience, *b* = −4.05 and 95% CI [−6.48, −1.62], but no relationship between intrusion or avoidance and resilience. Among men in the comparison group, there was no relationship between resilience or any subscale of traumatic stress: intrusion, avoidance, or arousal.

### Relationship Satisfaction at Follow-Up

Relationship satisfaction was similar among women in the study group and the comparison group, *t*(65) = 1.00, *p* = .32, and 95% CI [−5.77, 1.93]. Similarly, there was no difference in relationship satisfaction among men in the study group and the comparison group, *t*(50) = 0.81, *p* = .43, and 95% CI [−3.78, 4.53]. Within couples there was a significant correlation in satisfaction between partners, Pearson’s *r* = .34, *p* < .01, and no difference in satisfaction between women and men, *t*(50) = 1.63, *p* = .13, and 95% CI [−0.97, 6.97]. Pearson’s correlation between relationship satisfaction, resilience, and traumatic stress at T6 is shown in Table III.

**Table III.** Bivariate Pearson correlation of the combined data in the study and comparison groups between variables at Time 6 (10–12-year follow-up)

	1	2	3	4	5	6	7	8	9	10
Mothers										
1. Intrusion	–	.712**	.620**	−.380**	−.158					
2. Avoidance		–	.424**	−.393**	−.056					
3. Arousal			–	−.436**	−.128					
4. Resilience				–	.512**					
5. Relationship satisfaction					–					
Fathers										
6. Intrusion						–	.683**	.485**	−.201	−.071
7. Avoidance							–	.620**	−.288*	−.442*
8. Arousal								–	−.499**	.130
9. Resilience									–	.173
10. Relationship satisfaction										–

* *p* < .05;

** *p* < .01.

## Discussion

### Interpretation of Main Findings

The present study showed that having a child with a congenital malformation has long-term implications for parental wellbeing. Avoidance and arousal levels in the study group were higher at follow-up than shortly after diagnosis, indicating that parents experience considerable emotional strain over time. This can be understood according to the Model of Pediatric Medical Traumatic Stress (Kazak et al., 2006), which suggests that parental traumatic stress responses following a child’s medical event are often non-linear. For instance, we speculate whether a possible explanation for the increase in traumatic stress might be that the implications of diagnosis for development are more impactful later in childhood, compared with when the child is 6 weeks old (Harmsen et al., 2017). According to the model, this change could trigger a return of symptoms.

We did not observe any significant differences in resilience among parents in the study and comparison group. Rosenberg et al. (2014) found that parents of children with cancer reported lower resilience than population norms, indicating that the serious illness of a child may reduce parents’ overall resilience. In our study, parents appeared to retain these coping resources despite high levels of long-term traumatic stress. We did however observe an inverse relationship between resilience and traumatic stress in the study group. This may indicate that resilience resources could diminish among those parents who experience the greatest distress from the diagnosis. Theories of family resilience suggest that factors such as perceived support and cohesion are important in protecting individuals from traumatic stress symptoms following a child’s illness (Van Schoors et al., 2015). It is therefore plausible that those parents with less resilience experience greater distress over time. Alternatively, it may be that parents who reported lower resilience experience the diagnosis as more traumatic.

On average, men reported higher levels of resilience that women. Indeed, previous research has found that men tend to score higher on intrapersonal factors of resilience, while women tend to score higher on interpersonal factors (Friborg et al., 2003). Consistent with this finding, we found that men scored higher than women on intrapersonal factors, but in contrast, women did not score higher than men on the interpersonal factors. Despite increasing involvement of fathers in caregiving, studies continue to suggest that mothers still assume more responsibility for the daily needs of their children than fathers do (Brandth & Kvande, 2002). Moreover, in families of children with disability, the additional childcare burden often falls disproportionally on mothers (Öjmyr-Joelsson et al., 2009; Roach et al., 1999). For women, possibly an increased demand of caregiving might reduce the ability to seek out and maintain some of the social support resources they might otherwise have cultivated.

Relationship satisfaction was similar across groups and genders and most participants reported being satisfied with their partner. Partner support has been found to predict more positive adjustment to adversity in other contexts, such as a partner’s serious illness (Manne et al., 2004) or financial strain (Karademas & Roussi, 2017). Future studies should examine whether relationship satisfaction might moderate psychological adjustment to a child’s illness or congenital malformation and thus buffer some of the negative impact of detection of fetal anomaly.

It is interesting to note that there was a difference in the number of children at follow-up between women in the study and comparison group. At time of inclusion, there was no difference between the groups in terms of previous children, which suggests that women in the study group were less likely to have more children after the birth of a child with congenital malformation. That is despite women in the comparison group being older and more educated, which tend to be associated with having fewer children (Huber et al., 2010). This may, in part, be due to the increased practical and psychosocial demands of parenting a chronically ill child (Talley & Crews, 2007), which could impede parents’ desire for or self-perceived capacity to have more children. This highlights the multifaceted psychosocial impact of diagnosis of fetal anomaly for parents and the family unit (Cadman et al., 1991; Clarke-Stewart & Dunn, 2006).

### Strengths, Limitations, and Future Research Directions

The strengths of the present study include the prospective longitudinal design and use of well-validated standardized psychometric methods. To our knowledge, this is the first prospective study on psychological responses of parents to children with prenatal diagnosis of congenital malformation and unaffected parents, covering an extended follow-up period. Prospective enrollment of parents to a child with congenital malformation is resource demanding and long-term studies of this group are rare.

A limitation of the study is the heterogeneity of congenital malformations. Due to low prevalence of different malformations, we chose to include all major malformations in order to recruit a sizable sample. Research has found that parents to children with more severe and more ambiguous diagnoses tend to experience more distress; however, the greatest difference remains between parents with and without a diagnosis of congenital malformation (Skari et al., 2006). In order to examine a disease-specific prospective study, a much larger sample size would have been required.

Our study design is observational, and among women, the study group and the comparison group were not equal in terms of age, education, and number of children. However, the observed differences in sociodemographic factors may not necessarily affect associations between study variables (Nilsen et al., 2009; Wolke et al., 2009). Given the length of the study period, a number of participants were also lost to follow-up. This reduced the sample size at T6, which prevented a further examination of the role of sociodemographic variables due to limited statistical power. While attrition analysis found no significant differences among those who dropped out and those who remained, this inevitably raises the question of bias, as there could be differences in terms of variables that were not considered.

A strength of the study is the 10–12-year follow-up, but the lack of additional measurements between T5 and T6 is a major limitation. Future studies should further explore the period between these timepoints. In addition, resilience and relationship satisfaction were only measured at one timepoint and therefore these variables and their relationship to traumatic stress could not be examined longitudinally.

The inclusion of fathers as well as mothers fills an important gap in the existing literature (Hammarberg et al., 2008). However, in our study, all the partners were male, and the families were homogenous in terms of ethnicity and sexual orientation. There is a need for future research to explore parental stress experiences in more diverse samples to further our understanding of adjustment across families that hold various identities.

The results obtained here suggest that psychological support to parents who receive a diagnosis of fetal anomaly is important. Our findings showed that traumatic stress symptoms were inversely related to resilience. Resilience may be an important aspect to consider for future interventions aimed at improving psychosocial outcomes for families. This can be done either through working with the individual to increase their intrapersonal resilience resources, such as cognitive flexibility and self-efficacy (Burton et al., 2010), or by targeting interpersonal resilience resources. Interpersonal resources can be improved, for example, through increased social support, such as support groups (Ozbay et al., 2007). This may be a particularly salient target for interventions because many individuals can be reached at the same time. Parents to children with congenital anomalies frequently express a desire to participate in such groups (Marokakis et al., 2016).

## Conclusion

We found that the prenatal diagnosis of a fetal anomaly has implications for parental traumatic stress 10–12 years after the birth of the child. However, parents to children with congenital diagnoses reported resilience and relationship satisfaction at levels that were comparable to non-affected parents. This suggests that despite experiencing elevated distress over a long period, most parents to children with congenital malformations are able to maintain positive psychosocial resources. Knowing more about the protective factors that promote adaptation to diagnosis will be central in developing interventions aimed at improving psychosocial outcomes for families.

### Research Data Statement

The data that support the findings of this study are available in part upon request from the corresponding author [A.O.]. The data are not publicly available due to the information containing that could compromise the privacy of the research participants.

## Supplementary Data

Supplementary data can be found at: https://academic.oup.com/jpepsy.

## Funding

This work was supported by the Research Council of Norway (RCN; grant number 288083 and 301004), Norwegian Women’s Public Health Association, the Norwegian Association for Children with Congenital Heart Disease, University of Oslo, and Oslo University Hospital.

*Conflicts of interest*: None declared.

## Supplementary Material

jsac085_Supplementary_Data
