# Supplementary material for: Long-Term Impact of Diagnosed Fetal Anomaly on Parental Traumatic Stress, Resilience, and Relationship Satisfaction
Source: J Pediatr Psychol. 2022 Nov 18;48(2):181–92. doi: 10.1093/jpepsy/jsac085 (PMC9941832; doi:10.1093/jpepsy/jsac085)
Supplement: jsac085_Supplementary_Data [file jsac085_supplementary_data.docx]

**Supplementary materials**

**Table 4.** Linear mixed models of traumatic stress measured by the Impact of Events Scale (IES) subscales intrusion, avoidance and arousal among women and men measured at T1 (inclusion), T2 (2-3 weeks after T1), T3 (gestational age 30 weeks), T4 (gestational age 36 weeks), T5 (six weeks after birth), and T6 (ten-twelve years after birth).

|  | **Null model** | | | | **Model 1** | | | | **Model 2** | | | |
| --- | --- | --- | --- | --- | --- | --- | --- | --- | --- | --- | --- | --- |
|  | Estimate | St. Error | *t* | *p* | Estimate | St. Error | *t* | *p* | Estimate | St. Error | *t* | *p* |
| **Mothers** |  |  |  |  |  |  |  |  |  |  |  |  |
| **IES intrusion** |  |  |  |  |  |  |  |  |  |  |  |  |
| *Fixed effects* |  |  |  |  |  |  |  |  |  |  |  |  |
| Intercept | 11.16 | 0.56 | 19.88 | <.001 | 15.18 | 0.67 | 22.81 | <.001 | 22.92 | 0.90 | 25.41 | <.001 |
| Time  T2  T3  T4  T5  T6 |  |  |  |  | -3.99  -5.51  -5.10  -6.83  -3.19 | 0.67  0.63  0.63  0.62  0.85 | -6.34  -8.69  -8.10  -11.05  -3.74 |  | -5.50  -9.59  -10.18  -11.83  -8.74 | 0.96  1.02  0.97  0.92  1.30 | -5.70  -9.40  -10.50  -12.81  -6.74 |  |
| Group |  |  |  |  |  |  |  |  | -13.43 | 1.19 | -11.30 |  |
| Time:Group  T2:Group  T3:Group  T4:Group  T5:Group  T6:Group |  |  |  |  |  |  |  |  | 2.97  6.93  8.56  8.55  9.42 | 1.23  1.27  1.23  1.20  1.66 | 2.41  5.48  6.94  7.14  5.67 |  |
| *Random effects* | |  |  |  |  |  |  |  |  |  |  |  |
| Residual | 40.22 |  |  |  | 33.93 |  |  |  | 30.62 |  |  |  |
| σ^2^ | 6.34 |  |  |  | 5.83 |  |  |  | 5.53 |  |  |  |
| *Model inf* |  |  |  |  |  |  |  |  |  |  |  |  |
| AIC | 36358 |  |  |  | 6234 |  |  |  | 6103 |  |  |  |
| BIC | 6372 |  |  |  | 6272 |  |  |  | 6170 |  |  |  |
| Log likelihood | - 3176 |  |  |  | - 3109 |  |  |  | - 3037 |  |  |  |
| χ^2^(df) |  |  |  |  | 133.9 (5) |  |  |  | 143.1(6) |  |  |  |
| Pr(> Chisq) |  |  |  |  | <.001 |  |  |  | <.001 |  |  |  |
| **IES avoidance** |  |  |  |  |  |  |  |  |  |  |  |  |
| *Fixed effects* |  |  |  |  |  |  |  |  |  |  |  |  |
| Intercept | 4.39 | 0.40 | 10.91 | <.001 | 5.79 | 0.45 | 12.78 | <.001 | 10.34 | 0.61 | 16.87 | <.001 |
| Time  T2  T3  T4  T5  T6 |  |  |  |  | -1.80  -2.29  -2.52  -2.97  4.13 | 0.37  0.37  0.38  0.37  0.51 | -4.82  -6.06  -6.72  -8.06  8.12 |  | -2.58  -4.00  -3.93  -4.55  0.64 | 0.59  0.62  0.59  0.56  0.80 | -4.36  -6.40  -6.61  -8.05  0.81 |  |
| Group |  |  |  |  |  |  |  |  | -7.90 | 0.81 | -9.78 |  |
| Time:Group  T2:Group  T3:Group  T4:Group  T5:Group  T6:Group |  |  |  |  |  |  |  |  | 1.48  2.90  2.46  2.72  5.81 | 0.75  0.77  0.76  0.73  1.02 | 1.96  3.74  3.25  3.72  5.71 |  |
| *Random effects* |  |  |  |  |  |  |  |  |  |  |  |  |
| Residual | 15.92 |  |  |  | 11.98 |  |  |  | 11.44 |  |  |  |
| σ^2^ | 3.99 |  |  |  | 3.46 |  |  |  | 3.38 |  |  |  |
| *Model inf* |  |  |  |  |  |  |  |  |  |  |  |  |
| AIC | 5564 |  |  |  | 5357 |  |  |  | 5267 |  |  |  |
| BIC | 5579 |  |  |  | 5395 |  |  |  | 5334 |  |  |  |
| Log likelihood | - 2779 |  |  |  | - 2670 |  |  |  | - 2619 |  |  |  |
| χ^2^(df) |  |  |  |  | 217.5 (5) |  |  |  | 102.1 (6) |  |  |  |
| Pr(> Chisq) |  |  |  |  | <.001 |  |  |  | <.001 |  |  |  |
| **IES arousal** |  |  |  |  |  |  |  |  |  |  |  |  |
| *Fixed effects* |  |  |  |  |  |  |  |  |  |  |  |  |
| Intercept | 5.75 | 0.37 | 15.6 | <.001 | 7.23 | 0.43 | 16.82 | <.001 | 12.09 | 0.59 | 20.21 | <.001 |
| Time  T2  T3  T4  T5  T6 |  |  |  |  | -2.07  -2.18  -1.86  -3.07  1.89 | 0.39  0.39  0.39  0.38  0.53 | -5.33  -5.57  -4.77  -8.02  3.58 |  | -3.66  -5.04  -5.15  -6.58  -2.35 | 0.59  0.62  0.59  0.56  0.79 | -6.21  -8.08  -8.69  -11.66  -2.96 |  |
| Group |  |  |  |  |  |  |  |  | -8.41 | 0.79 | -10.68 |  |
| Interaction  T2:Group  T3:Group  T4:Group  T5:Group  T6:Group |  |  |  |  |  |  |  |  | 2.91  4.85  5.58  6.00  7.14 | 0.75  0.77  0-75  0.73  1.02 | 3.88  6.26  7.40  8.20  7.03 |  |
| Random effects |  |  |  |  |  |  |  |  |  |  |  |  |
| Residual | 15.10 |  |  |  | 12.94 |  |  |  | 11.42 |  |  |  |
| σ^2^ | 3.86 |  |  |  | 3.60 |  |  |  | 3.34 |  |  |  |
| AIC | 5492 |  |  |  | 5381 |  |  |  | 5252 |  |  |  |
| BIC | 5507 |  |  |  | 5420 |  |  |  | 5319 |  |  |  |
| Log likelihood | -2743 |  |  |  | -2682 |  |  |  | -2612 |  |  |  |
| χ^2^(df) |  |  |  |  | 120.8 (5) |  |  |  | 141.6 (6) |  |  |  |
| Pr(> Chisq) |  |  |  |  | <.001 |  |  |  | <.001 |  |  |  |
| **Fathers** |  |  |  |  |  |  |  |  |  |  |  |  |
| **IES intrusion** |  |  |  |  |  |  |  |  |  |  |  |  |
| *Fixed effects* |  |  |  |  |  |  |  |  |  |  |  |  |
| Intercept | 11.16 | 0.56 | 19.88 | <.001 | 10.50 | 0.55 | 19.14 | <.001 | 15.44 | 0.79 | 19.49 | <.001 |
| Time  T2  T3  T4  T5  T6 |  |  |  |  | -3.18  -4.64  -4.75  -4.67  0.95 | 0.50  0.51  0.52  0.50  0.73 | -6.32  -9.09  -9.19  -9.31  1.31 |  | -4.27  -7.88  -7.83  -7.90  -1.52 | 0.82  0.87  0.83  0.78  1.28 | -5.19  -9.01  -9.42  -10.08  -1.18 |  |
| Group |  |  |  |  |  |  |  |  | -8.38 | 1.03 | -8.12 |  |
| Interaction  T2:Group  T3:Group  T4:Group  T5:Group  T6:Group |  |  |  |  |  |  |  |  | 2.13  5.19  5.06  5.40  4.15 | 1.02  1.06  1.04  1.00  1.54 | 2.09  4.88  4.86  5.40  2.69 |  |
| *Random effects* |  |  |  |  |  |  |  |  |  |  |  |  |
| Residual | 40.22 |  |  |  | 18.57 |  |  |  | 17.44 |  |  |  |
| σ^2^ | 6.34 |  |  |  | 4.31 |  |  |  | 4.18 |  |  |  |
| *Model inf* |  |  |  |  |  |  |  |  |  |  |  |  |
| AIC | 6358 |  |  |  | 4763 |  |  |  | 4698 |  |  |  |
| BIC | 6372 |  |  |  | 4800 |  |  |  | 4763 |  |  |  |
| Log likelihood | -3176 |  |  |  | -2373 |  |  |  | -2335 |  |  |  |
| χ^2^(df) |  |  |  |  | 154.7(5) |  |  |  | 76.63(6) |  |  |  |
| Pr(> Chisq) |  |  |  |  | <.001 |  |  |  | <.001 |  |  |  |
| **IES avoidance** |  |  |  |  |  |  |  |  |  |  |  |  |
| *Fixed effects* |  |  |  |  |  |  |  |  |  |  |  |  |
| Intercept | 2.96 | 0.28 | 10.41 | <.01 | 3.92 | 0.33 | 11.71 | <.01 | 7.11 | 0.47 | 15.04 | <.001 |
| Time  T2  T3  T4  T5  T6 |  |  |  |  | -1.39  -1.80  -2.01  -2.19  5.91 | 0.30  0.30  0.31  0.30  0.43 | -4.65  -5.96  -6.53  -7.34  13.64 |  | -2.24  -3.26  -3.63  -4.08  4.43 | 0.49  0.52  0.49  0.47  0.77 | -4.56  -6.24  -7.33  -8.71  5.76 |  |
| Group |  |  |  |  |  |  |  |  | -5.41 | 0.62 | -8.79 |  |
| Interaction  T2:Group  T3:Group  T4:Group  T5:Group  T6:Group |  |  |  |  |  |  |  |  | 1.57  2.42  2.71  3.16  2.45 | 0.61  0.64  0.62  0.60  0.92 | 2.56  3.81  4.34  5.29  2.66 |  |
| *Random effects* |  |  |  |  |  |  |  |  |  |  |  |  |
| Residual | 11.12 |  |  |  | 6.53 |  |  |  | 6.24 |  |  |  |
| σ^2^ | 3.33 |  |  |  | 2.56 |  |  |  | 2.50 |  |  |  |
| *Model inf* |  |  |  |  |  |  |  |  |  |  |  |  |
| AIC | 4285 |  |  |  | 3980 |  |  |  | 3914 |  |  |  |
| BIC | 4299 |  |  |  | 4017 |  |  |  | 3979 |  |  |  |
| Log likelihood | -2140 |  |  |  | -1982 |  |  |  | -1943 |  |  |  |
| χ^2^(df) |  |  |  |  | 315.6(5) |  |  |  | 78.2(6) |  |  |  |
| Pr(> Chisq) |  |  |  |  | <.001 |  |  |  | <.001 |  |  |  |
| **IES arousal** |  |  |  |  |  |  |  |  |  |  |  |  |
| *Fixed effects* |  |  |  |  |  |  |  |  |  |  |  |  |
| Intercept | 3.46 | 0.27 | 12.96 | <.001 | 3.94 | 0.31 | 12.55 | <.001 | 6.47 | 0.45 | 14.27 | <.001 |
| Time  T2  T3  T4  T5  T6 |  |  |  |  | -1.02  -1.37  -1.29  -0.80  5.41 | 0.28  0.28  0.29  0.28  0.40 | -3.67  -4.87  -4.53  -2.89  13.47 |  | -1.37  -2.72  -3.11  -1.94  3.98 | 0.46  0.49  0.46  0.43  0.71 | -2.99  -5.59  -6.72  -4.46  5.56 |  |
| Group |  |  |  |  |  |  |  |  | -4.29 | 0.59 | -7.26 |  |
| Interaction  T2:Group  T3:Group  T4:Group  T5:Group  T6:Group |  |  |  |  |  |  |  |  | 0.76  2.18  2.94  1.93  2.29 | 0.57  0.59  0.58  0.56  0.86 | 1.33  3.69  5.06  3.46  2.67 |  |
| Random effects |  |  |  |  |  |  |  |  |  |  |  |  |
| Residual | 5.62 |  |  |  | 5.62 |  |  |  | 5.40 |  |  |  |
| σ^2^ | 2.37 |  |  |  | 2.37 |  |  |  | 2.33 |  |  |  |
| AIC | 4119 |  |  |  | 3871 |  |  |  | 3819 |  |  |  |
| BIC | 4133 |  |  |  | 3908 |  |  |  | 3884 |  |  |  |
| Log likelihood | -2056 |  |  |  | -1927 |  |  |  | -1895 |  |  |  |
| χ^2^(df) |  |  |  |  | 258.8(5) |  |  |  | 63.46(6) |  |  |  |
| Pr(> Chisq) |  |  |  |  | <.001 |  |  |  | <.001 |  |  |  |
